# Mutations in the pH-Sensing G-protein-Coupled Receptor *GPR68* Cause Amelogenesis Imperfecta

**DOI:** 10.1016/j.ajhg.2016.08.020

**Published:** 2016-09-29

**Authors:** David A. Parry, Claire E.L. Smith, Walid El-Sayed, James A. Poulter, Roger C. Shore, Clare V. Logan, Chihiro Mogi, Koichi Sato, Fumikazu Okajima, Akihiro Harada, Hong Zhang, Mine Koruyucu, Figen Seymen, Jan C.-C. Hu, James P. Simmer, Mushtaq Ahmed, Hussain Jafri, Colin A. Johnson, Chris F. Inglehearn, Alan J. Mighell

**Affiliations:** 1 Leeds Institute of Biomedical and Clinical Sciences, University of Leeds, St. James’s University Hospital, Leeds LS9 7TF, UK; 2 Centre for Genomic and Experimental Medicine, Institute of Genetics and Molecular Medicine, University of Edinburgh, Western General Hospital, Edinburgh EH4 2XU, UK; 3 School of Dentistry, Department of Oral Biology, St. James’s University Hospital, University of Leeds, Leeds LS9 7TF, UK; 4 Oral Biology Department, College of Dentistry, Suez Canal University, El Salam District, Ismaileya 41611, Egypt; 5 Oral Biology Department, College of Dentistry, Gulf Medical University, Al Jurf, Ajman 3787, United Arab Emirates; 6 Laboratory of Signal Transduction, Institute for Molecular and Cellular Regulation, Gunma University, Maebashi 371-8512, Japan; 7 Department of Cell Biology, Graduate School of Medicine, Osaka University, Osaka 565-0871, Japan; 8 Departments of Biologic and Materials Sciences, University of Michigan School of Dentistry, 1210 Eisenhower Place, Ann Arbor, MI 48108, USA; 9 Department of Pedodontics, Faculty of Dentistry, Istanbul University, Istanbul 34116, Turkey; 10 Clinical Genetics, Leeds Teaching Hospitals NHS Trust, Chapel Allerton Hospital, Leeds LS7 4SA, UK; 11 Gene Tech Lab 146/1, Shadman Jail Road, Lahore 54000, Pakistan

**Keywords:** Amelogenesis imperfecta, GPR68, amelogenesis, teeth, pH sensing

## Abstract

Amelogenesis is the process of dental enamel formation, leading to the deposition of the hardest tissue in the human body. This process requires the intricate regulation of ion transport and controlled changes to the pH of the developing enamel matrix. The means by which the enamel organ regulates pH during amelogenesis is largely unknown. We identified rare homozygous variants in *GPR68* in three families with amelogenesis imperfecta, a genetically and phenotypically heterogeneous group of inherited conditions associated with abnormal enamel formation. Each of these homozygous variants (a large in-frame deletion, a frameshift deletion, and a missense variant) were predicted to result in loss of function. *GPR68* encodes a proton-sensing G-protein-coupled receptor with sensitivity in the pH range that occurs in the developing enamel matrix during amelogenesis. Immunohistochemistry of rat mandibles confirmed localization of GPR68 in the enamel organ at all stages of amelogenesis. Our data identify a role for GPR68 as a proton sensor that is required for proper enamel formation.

## Main Text

The formation of dental enamel (amelogenesis) is a process of biomineralization taking years to complete in the human dentition and resulting in the deposition of the hardest, most mineralized tissue in the body. Mature enamel consists of highly organized calcium hydroxyapatite (Ca_10_[PO_4_]_6_[OH]_2_) crystals, which form in a discrete extracellular compartment within the developing tooth.^1^ Ameloblasts, the enamel-forming cells, regulate the mineralization of enamel by secreting matrix proteins that act as modulators of crystal deposition and growth. They exert temporo-spatial control over protease secretion to process and degrade matrix proteins, remove degraded protein from the matrix, and control mineral ion transport to accommodate crystal growth.^2^ Amelogenesis is accomplished in two stages. During the secretory stage, thin mineral ribbons separated by organic matrix initiate at the dentin surface and grow in length until the enamel layer reaches full thickness. During the maturation stage, the crystal ribbons deposited during the secretory stage expand in width and thickness as the organic matrix is degraded and reabsorbed.

The formation of hydroxyapatite crystals results in the acidification of the surrounding environment; up to 14 moles of protons are produced per mole of apatite formed.^1^ Although secretory-stage enamel contains a large volume of matrix proteins that might provide buffering capacity, during the maturation stage these proteins are degraded, and the rate of mineralization is at its highest. Therefore, at the time of greatest proton generation, the buffering capacity of enamel is at its lowest.^3^ Maturation-stage enamel has alternating regions of higher and lower pH that coincide with “ruffle-ended” or “smooth-ended” morphologies of the overlying ameloblasts, respectively. Multiple anion exchangers (bicarbonate and chloride exchangers) and H^+^-ATPase proton pumps are believed to contribute to pH changes.^4^, ^5^ However, the mechanisms by which ameloblasts sense and respond to the pH changes of the underlying enamel are as yet obscure.

Amelogenesis imperfecta (AI [MIM: 104500]) refers to a genetically and phenotypically heterogeneous group of inherited conditions associated with the formation of abnormally thin, soft, or brittle enamel. Genes associated with non-syndromic AI encode proteins involved in the formation and maintenance of the developing enamel matrix (including *AMELX*^6^ [MIM ^∗^300391], *ENAM*^7^ [MIM: 606585], *KLK4*^8^ [MIM: 603767], *MMP20*^9^ [MIM: 604629], *FAM20A*^10^ [MIM: 611062], *C4orf26*^11^ [MIM: 614829] and *AMBN*^12^ [MIM: 601259]), ion transport (*SLC24A4*^13^ [MIM: 609840]), extracellular matrix adhesion (*LAMB3*^14^, ^15^ [MIM: 150310], *ITGB6*^16^, ^17^ [MIM: 147558], *COL17A1*^18^ [MIM: 113811], and *LAMA3*^19^ [MIM: 600805]) and proteins associated with intracellular vesicles (*FAM83H*^20^ [MIM: 611927] and *WDR72*^21^ [MIM: 613214]).

**Figure 1:**
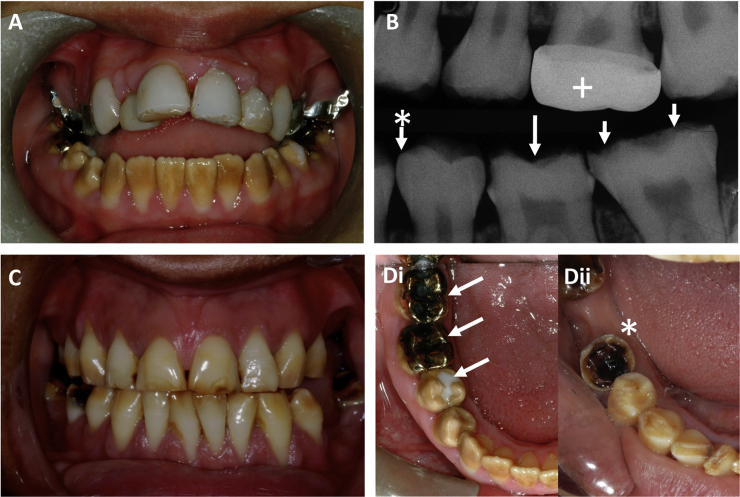
Clinical Appearances of the Dentition in Individuals with *GPR68* Mutations The clinical phenotype is consistent with hypomaturation AI. (A) The lower arch permanent dentition of the proband at 15 years old is characterized by opaque, discolored enamel that in part is due to extrinsic staining. The upper dentition has been restored. The marked anterior open bite was not observed in other affected family members. (B) An intraoral radiograph of the proband at 14 years old illustrates a near-normal enamel morphology in the recently erupted second premolar tooth (arrow with asterisk) but premature failure of enamel with tooth substance loss in the permanent molar teeth (arrows), which have been present in the mouth longer and have been subject to the greatest functional load. A crowned upper tooth is marked with a cross. (C) The dentition of an affected sibling at 20 years old is characterized by less-extrinsic staining but clear attrition along the occlusal plane and failure of posterior teeth, several of which are missing. (D) Comparative occlusal views of the lower right quadrants in the proband (i) and sibling (ii) confirm the similarities in enamel appearances and the more-extensive attrition in the older individual, whose anterior teeth occlude. Restorations are marked with arrows, and a grossly broken-down tooth is marked with an asterisk.

We identified a UK consanguineous family (AI-5) that originated from the Mirpur region of Pakistan and had some family members affected by autosomal-recessive hypomineralized AI. Permanent and deciduous enamel were abnormally opaque in appearance and prone to early functional failure, but affected individuals did not show obvious signs of any other health problems (Figure 1 and Figure S3). This study was performed in accordance with the principles of the declaration of Helsinki, with informed individual consent and ethical approval.

Peripheral blood samples were obtained from affected and unaffected family members, and genomic DNA was prepared by a conventional salting out method. Affymetrix 10K SNP chip analysis of affected DNA indicated a 13.1 Mb homozygous region on chromosome 14q between SNPs rs1241903 and rs722869. LINKMAP^22^ multipoint linkage analysis of microsatellite markers D14S1052, D14S1015, and D14S553 versus disease confirmed linkage with a maximum LOD score of 3.1 at marker D14S1015 and refined the disease region to an 11.8 Mb locus containing 65 protein-coding RefSeq genes between rs1241903 and D14S996 (Figure S1 and Table S1 in the Supplemental Data available online).

**Figure 2:**
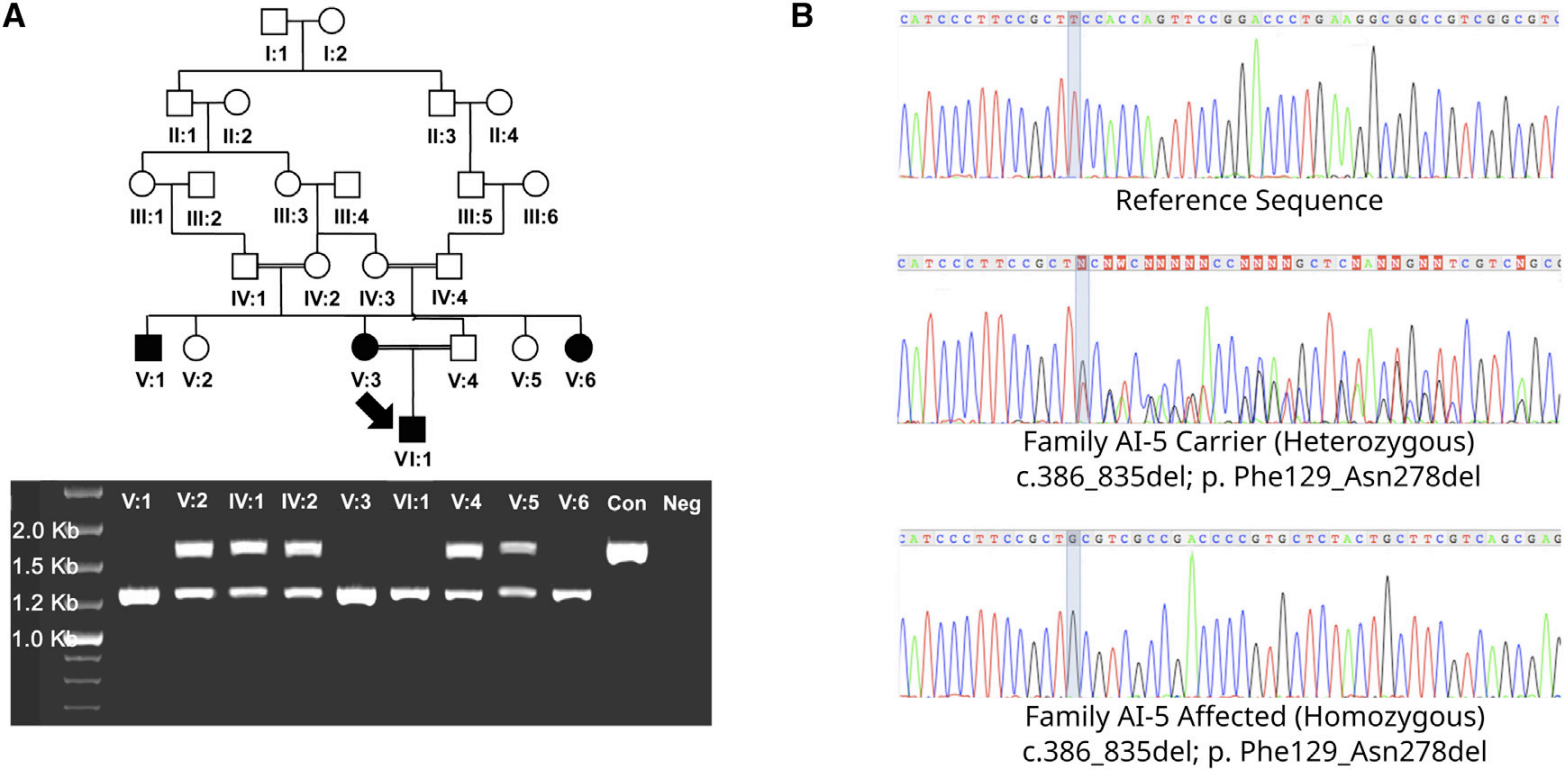
Identification of a *GPR68* Deletion in Family AI-5 (A) Segregation of a deletion in *GPR68* with amelogenesis imperfecta in family AI-5. The coding sequence of *GPR68* was amplified by PCR to produce a 1,685 bp product in control DNA (Con). All affected AI-5 family members for whom DNA was available were homozygous for a 450 bp deletion, whereas unaffected carriers were heterozygous for this deletion. Neg; negative control. (B) Electropherograms of *GPR68* genomic DNA sequence showing the homozygous c.386_835del (GenBank: NM_001177676.1) deletion in an affected individual, the same mutation in a heterozygous state in a carrier, and normal sequence from control DNA.

We considered two genes within the linked region to be strong candidates for involvement in the disease: *CALM1* (MIM: 114180) and *GPR68* (MIM: 601404). Calmodulin 1 (*CALM1*) has been localized to ameloblasts and might help to regulate calcium transport,^23^, ^24^ but direct sequencing of all *CALM1* exons in affected individuals failed to reveal any mutation. We then screened *GPR68*, which has been identified as a proton-sensing G-protein-coupled receptor (GPCR)^25^ implicated in osteoblast^25^, ^26^, ^27^ and osteoclast function.^28^, ^29^ Size fractionation by agarose gel electrophoresis and direct sequencing revealed an in-frame 450 bp homozygous deletion in affected individuals (Figure 2 and Figure S2) in the sole coding exon of *GPR68* (GenBank: NM_001177676.1 [c.386_835del (p.Phe129_Asn278del)]). The deletion segregated with the disease phenotype in the family (Figure 2A and Figure S2) and was absent in 170 ethnically matched control individuals. This mutation deletes four of the seven transmembrane helices and removes three of the six histidine residues previously shown to be crucial to the pH sensitivity or structural integrity of the protein.^25^ Any protein made is almost certain to lack normal GPR68 function and could be unstable.

After mapping the chromosome 14 locus in family AI-5, we checked for mutations in *SLC24A4*, which lies in the linkage region and was previously identified as a cause of AI.^13^ Screening of all coding regions and flanking intronic sequences failed to identify any sequence variants in affected members of AI-5; however, we did not rule out regulatory or deep intronic mutations. Moreover, exome sequencing of individual VI:1 identified only one further rare (<1% allele frequency) variant at this locus, a missense change in *SERPINA12* (rs192558870 [GenBank: NM_173850.3: c.656A>G [p.Asp219Gly]). *SERPINA12* encodes an adipokine that increases insulin sensitivity, and a common nonsense variant (rs61757459) was identified in data from ExAC,^30^ suggesting that variation in *SERPINA12* is not a likely cause of AI.

**Figure 3:**
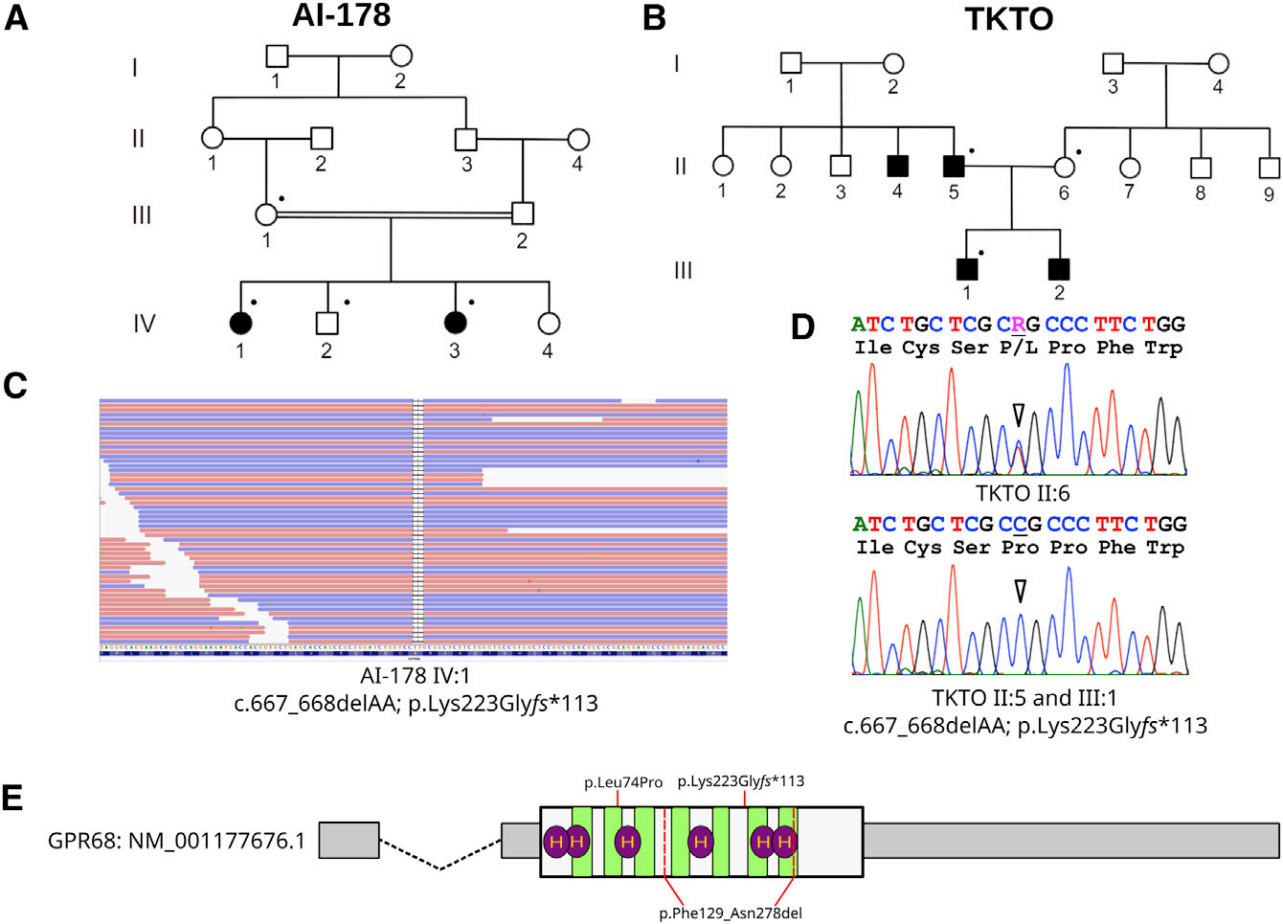
Identification of *GPR68* Mutations in Two Additional Families Affected by Amelogenesis Imperfecta (A and B) Pedigrees of family AI-178 (A) and family TKTO (B). Dots indicate family members for whom DNA samples were available. (C) IGV snapshot of the frameshift mutation identified in family AI-178. (D) Sanger traces of the missense mutation identified in family TKTO. (E) Schematic depiction of the *GPR68* transcript and encoded protein features. UTR regions are shown in thin gray boxes, introns are indicated by dashed lines, and the coding region is shown by a taller white box. Light green regions indicate transmembrane helices, and purple ovals denote histidine residues shown to be essential for the normal pH-sensing function or structural integrity of GPR68.^16^ Variants identified in this study are marked in red with the associated protein consequences.

Sanger sequencing of *GPR68* and analysis of exome sequencing data in 80 AI families identified two additional families in which some members harbored putative disease-causing variants in *GPR68*. In family AI-178 (Figure 3A and Figure S4), of Pakistani heritage, we identified a homozygous frameshift deletion (c.667_668delAA [p.Lys223Gly*fs*^∗^113]) expected to remove two of the encoded protein’s transmembrane helices and two of the pH-sensing histidine residues (Figures 3C and 3E). Any protein produced is likely to lack the physiological function of the wild-type protein. In family TKTO (Figure 3B and Figures S5 and S6), of Turkish heritage, exome sequencing identified a homozygous missense mutation (c.221T>C [p.Leu74Pro]) consistent with unrecorded consanguinity, altering a residue in the second transmembrane helix of GPR68 (Figures 3D and 3E). The mutation in family TKTO was predicted to be damaging by PolyPhen2,^31^ which gave a score of 1.0 under the HVAR model. The altered residue is fully conserved in GPR68 orthologs and shows strong conservation in the proton-sensing GPCRs GPR4, GPR65, and GPR132 (Figure S7). Proline residues are often found in loops at the end of alpha helices in globular proteins and as alpha helix breakers in transmembrane helices. However, the replacement of a highly conserved leucine residue with a proline immediately adjacent to another proline residue (Pro75) was considered likely to destabilize the secondary structure of the second transmembrane helix of GPR68 and severely alter the functioning of the protein. We confirmed familial segregation of these variants with AI for all individuals for whom DNA was available (Figures 3A, 3B, and 3D and Figure S5). No co-segregating health problems, including bone conditions, were evident from review of the clinical information available for the three families.

All three variants identified in *GPR68* as putative causes of AI were confirmed to be absent from public databases, including dbSNP, EVS, and ExAC. ExAC contains a large cohort (8,256) of South Asian samples, so absence of the frameshift identified in family AI-178 and the missense variant identified in family TKTO would suggest that these are not common polymorphisms in the populations from which these families originate, but rather that they are very rare or private alleles. Because the 450 bp deletion identified in AI-5 is unlikely to be detected by the methods employed by ExAC, we confirmed the absence of this variant by using agarose gel electrophoresis in 170 ethnically matched control samples. Exome sequencing data of affected individuals from all three families were analyzed to confirm that no mutation could be identified in genes previously implicated in AI.

Enamel formation requires strict regulation of ion transport and extracellular-matrix processing. Both crystal growth^32^ and protease activity^33^, ^34^ are sensitive to extracellular pH, and the need for a pH-sensing system during amelogenesis was proposed almost two decades ago.^2^ During the secretory stage of amelogenesis, long, thin crystals are embedded in a self-assembled extracellular matrix. During the transition and early maturation stage, this protein scaffold is degraded primarily by the proteases MMP20 and KLK4 and mostly removed from the tissue.^35^ Maturation-stage enamel is therefore porous and has fluid-filled intercrystalline spaces.^36^ Transport of calcium and phosphate ions into the matrix then results in secondary crystal growth, where the enamel crystals grow in both width and thickness, eventually occluding almost the entire tissue volume and ultimately generating the hardest and most highly mineralized tissue of the skeleton.^2^ During the maturation stage of amelogenesis, ameloblasts undergo cyclic changes in cell morphology between ruffle-ended ameloblasts (RAs), in which the cells form tight junctions and have membrane invaginations at their apical membranes, and leaky smooth-ended ameloblasts (SAs), in which the cells lack the apical tight junctions and ruffled morphology. Areas of enamel covered by RAs are mildly acidic (pH 6.1–6.8), whereas SAs cover areas of near physiological pH (pH 7.2–7.4).^33^, ^37^ The switching between RAs, which allow the build-up of protons in the developing enamel and might even pump out protons from their apical surface,^5^ and SAs, which allow release of bicarbonate ions into the developing enamel,^4^ permits a pH cycling that is critical for the degradation^38^ and removal of matrix proteins and the continued growth of hydroxyapatite crystals. Coordinated switching between RA and SA cell morphologies is likely to be dependent upon a pH-sensing mechanism.

GPR68 is a recognized pH sensor in osteoblasts and osteocytes. Histidine residues situated on the externally facing domain of the protein help it to sense pH between 7.8 (completely inactive) and 6.8 (fully active).^25^ GPR68 activation leads to inositol phosphate formation and release of calcium from intracellular stores^25^, ^39^ and is therefore a good candidate for the role of pH sensor in the enamel organ. Interestingly, inositol phosphate release is associated with cytoplasmic reorganization^40^ (an absolute requirement for the switch from RAs to SAs) and even with membrane ruffling,^41^ as seen in RAs. Furthermore, a recent study has demonstrated that overexpression of GPR68 in Caco-2 cells results in increased barrier formation upon acidification of the environment,^42^ and another study has shown that GPR68 signaling regulates the expression of Na^+^/H^+^ antiporters and H^+^-ATPase transporters in epithelial cells.^43^ Both of these functions are potentially relevant to our proposed role for GPR68 in amelogenesis.

**Figure 4:**
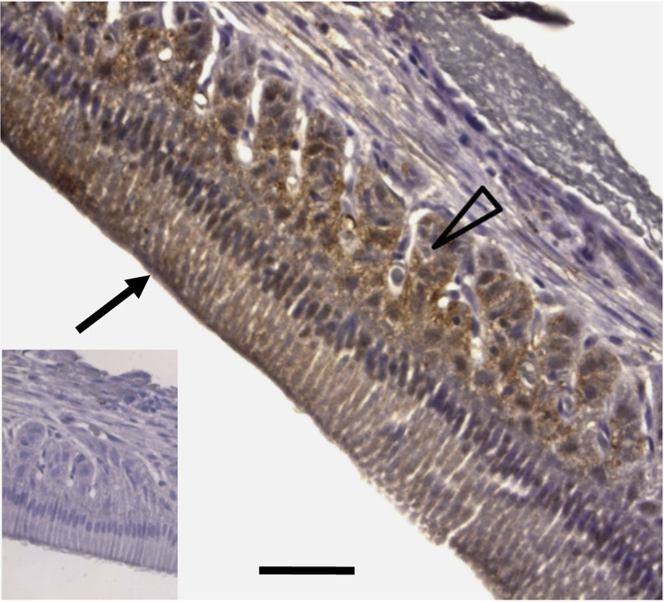
GPR68 localization during Rat Incisor Development Use of a previously characterized antibody^25^, ^29^ (Lifespan Biosciences, LS-A1194) allowed observation of GPR68 immunoreactivity in the enamel organ throughout amelogenesis. Staining is visible in the stellate reticulum (open arrow head) and ameloblasts (arrow) at the secretary stage. The panel inset shows a negative control where the primary antibody has been omitted. The scale bar represents 100 μm.

In order to confirm GPR68 localization in the developing tooth, we performed immunohistochemistry on sections of demineralised rat mandible. GPR68 immunoreactivity was observed in the enamel organ, including the ameloblast cells, during all stages of amelogenesis (Figure 4), consistent with a role in enamel formation. Prominent staining of the apical surface of ameloblasts with anti-GPR68 is consistent with a role for GPR68 as a pH monitor of the developing enamel matrix. High levels of staining within the papillary layer is also consistent with the suggestion that the ameloblasts and papillary layer are acting in concert as a functional unit.^4^, ^44^, ^45^

*Gpr68* (*Ogr1*)-knockout mice have been described previously,^46^ but no enamel defects were noted. We investigated the incisors of knockout mice and wild-type littermates to determine whether these mice might provide a useful model for AI. Transverse microradiography and energy-dispersive X-ray spectroscopy analyses did not reveal differences between the incisors of knockout mice and wild-type littermates, as might have been expected if the teeth of *Ogr1*-null mice reflected the phenotypes for the families presented (data not shown). However, scanning electron microscopy did reveal a more subtle change, involving possible retardation in the formation of, and alteration in the structure of, incisor enamel in knockout animals (Figure S8). Furthermore, developmentally, there appears to be a delay in the normal yellowing^47^, ^48^ of the maxillary incisor in the *Ogr1*-null mice (Figure S9). The lack of a clear enamel phenotype in rodent incisors might be due to the timing differences between human and mouse amelogenesis. Enamel maturation in the human permanent dentition takes many months or even years, whereas in the continually erupting incisors of mice the enamel matures in a matter of days. The genetic background of the mice used could also be an important factor. Mice null for the bicarbonate transporter *Slc4a2* were observed either to completely lack teeth^49^ or to have hypomineralized enamel^50^ in separate experiments involving animals of differing strains.

Our data suggest that GPR68 fulfils an essential role during amelogenesis in humans but that this function is not so crucial in mice. We propose a physiological function for GPR68 as a pH sensor and potential RA/SA switch during enamel formation, which could be confirmed by functional investigations and help elucidate mechanisms of pH regulation during amelogenesis.

## Accession Numbers

The variants reported in this paper have been deposited into the ClinVar database at the National Centre for Biotechnology Information under accession numbers SCV000298218–SCV000298220.

## Web Resources

Combined Annotation Dependent Depletion, http://cadd.gs.washington.edu/info
dbSNP, http://www.ncbi.nlm.nih.gov/projects/SNP/
ExAC, http://exac.broadinstitute.org
Exome Variant Server, http://evs.gs.washington.edu/EVS/
OMIM, http://www.omim.org/
PolyPhen2 http://genetics.bwh.harvard.edu/pph2/
SIFT, http://sift.jcvi.org/

## Supplemental Data

Document S1. Figures S1–S9 and Tables S2–S4

Table S1. SNP Genotypes for AI-5 Individuals for the Chromosome 14 Locus The disease region is highlighted in blue.

Document S2. Article plus Supplemental Data
