# Supplementary material for: Mutations in the pH-Sensing G-protein-Coupled Receptor GPR68 Cause Amelogenesis Imperfecta
Source: Am J Hum Genet. 2016 Sep 29;99(4):984–90. doi: 10.1016/j.ajhg.2016.08.020 (PMC5065684; doi:10.1016/j.ajhg.2016.08.020)
Supplement: Document S1. Figures S1–S9 and Tables S2–S4 [file mmc1.pdf]

The American Journal of Human Genetics, Volume *99*

Supplemental Data

Mutations in the pH-Sensing G-protein-Coupled Receptor *GPR68* Cause Amelogenesis Imperfecta

David A. Parry, Claire E.L. Smith, Walid El-Sayed, James A. Poulter, Roger C. Shore, Clare V. Logan, Chihiro Mogi, Koichi Sato, Fumikazu Okajima, Akihiro Harada, Hong Zhang, Mine Koruyucu, Figen Seymen, Jan C.-C. Hu, James P. Simmer, Mushtaq Ahmed, Hussain Jafri, Colin A. Johnson, Chris F. Inglehearn, and Alan J. Mighell

Supplemental Data

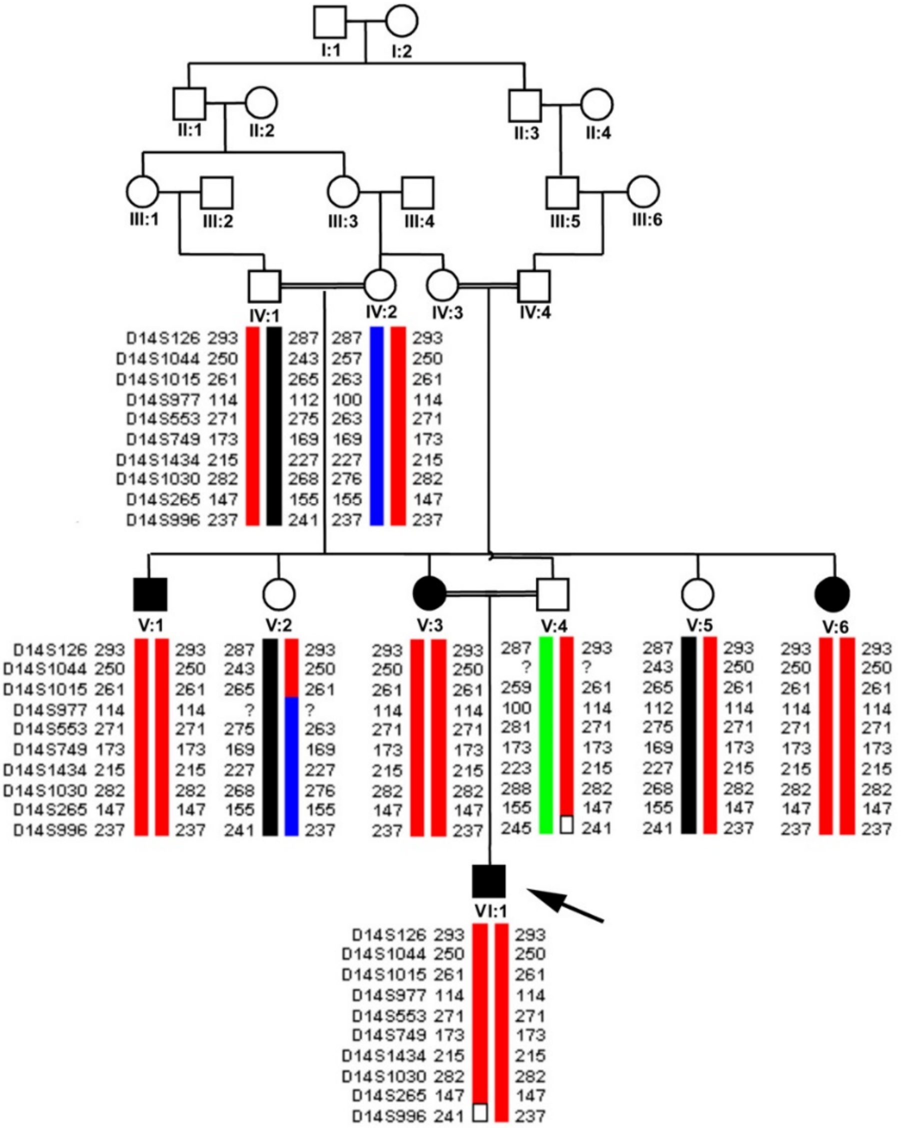

Figure S1. Chromosome 14 haplotypes in family AI-5. Microsatellite genotypes for all available family members. The disease haplotype is shown in red.

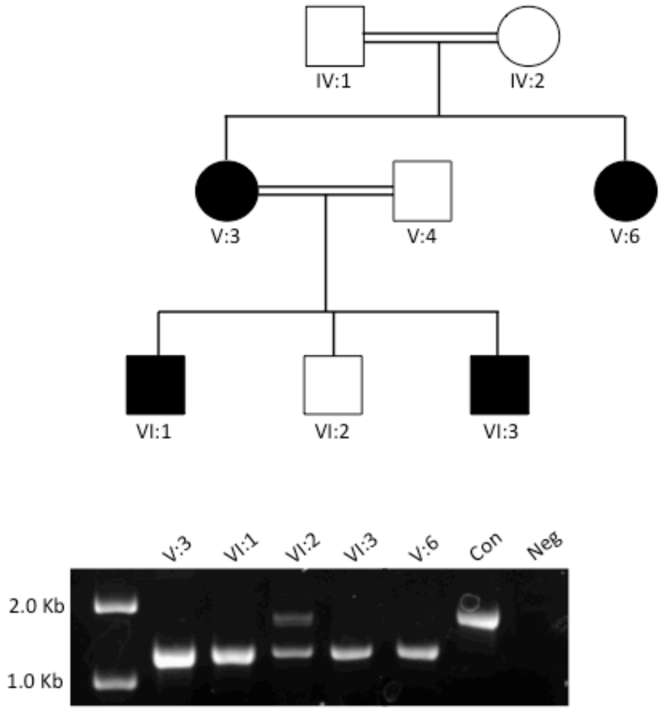

Figure S2. Segregation analysis for AI-5 family members VI:2 and VI:3. Additional segregation of a deletion in *GPR68* with Amelogenesis Imperfecta in family AI-5. DNA for individuals VI:2 and VI:3 was obtained at a later date to DNA for other AI-5 family members and therefore segregation analysis is shown separately to that shown in Figure 2. The coding sequence of *GPR68* was amplified by PCR to produce a 1685 bp product in control DNA (Con). All affected members of family AI-5 for whom DNA was available were homozygous for a 450 bp deletion while unaffected carriers were heterozygous for this deletion. Neg; negative control.

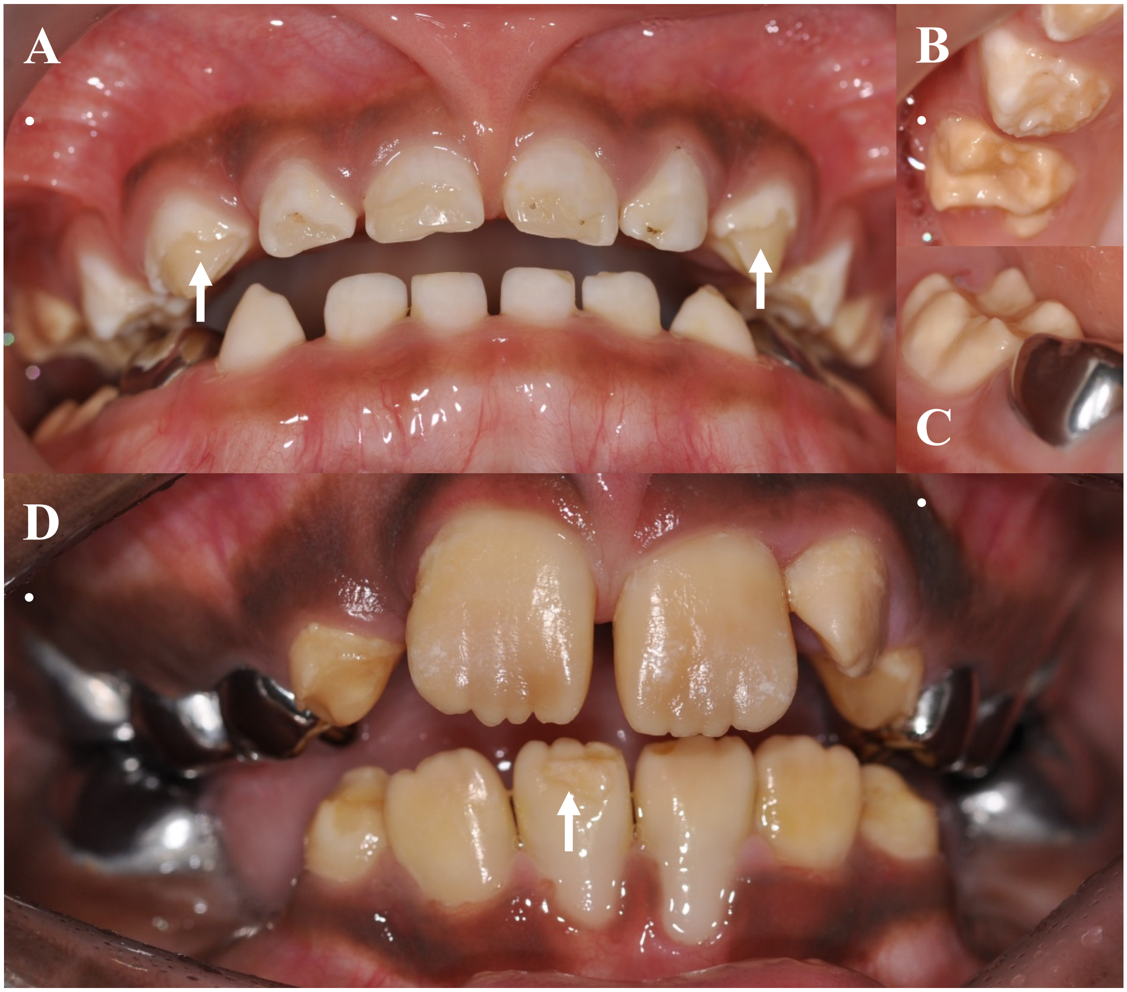

Figure S3. Further dental phenotype images for family AI-5. *A-C*. Primary dentition of VI:3 (only included in supplemental pedigree Figure S2) illustrating early loss of opaque enamel due to fracturing (examples marked with arrows in *A*). The upper (*B*.) and lower (*C*.) first permanent molar teeth have a creamier appearance without any gross loss of enamel. *D*. The anterior secondary dentition in VI:1 soon after eruption exhibit the typical creamy opaque appearance that tend to darken over time with some premature loss of enamel (arrow).

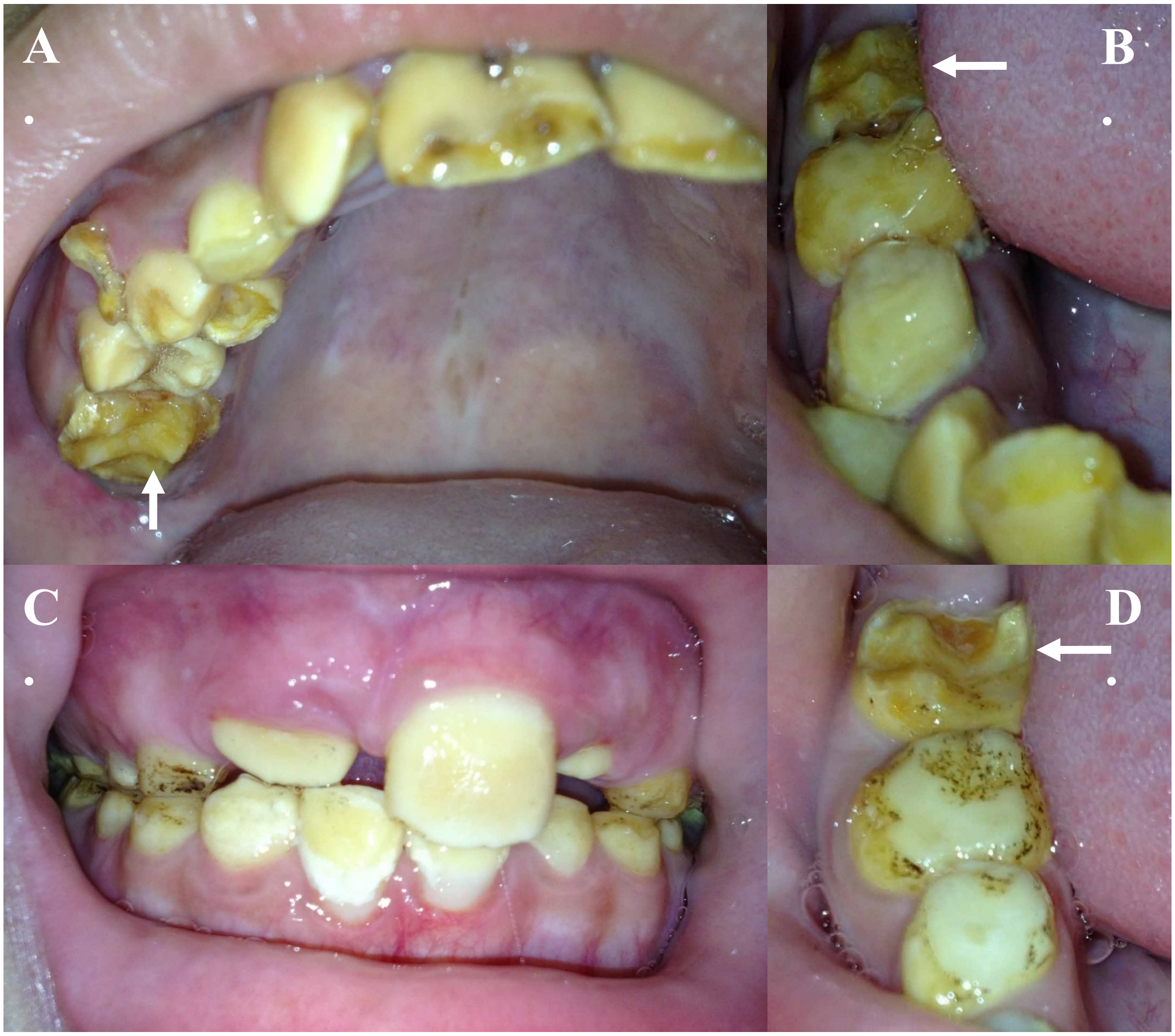

Figure S4. Dental phenotype images for family AI-178. *A & B*: Primary dentition of IV:3 illustrating the typical features of opaque enamel that prematurely fractures. Upper (*A.*) and lower (*B.*) first permanent molar teeth are marked with arrows. (*C & D*) The early mixed dentition of IV:1 as the upper central incisor teeth erupt with early loss of enamel in the lower right first permanent molar tooth (arrow).

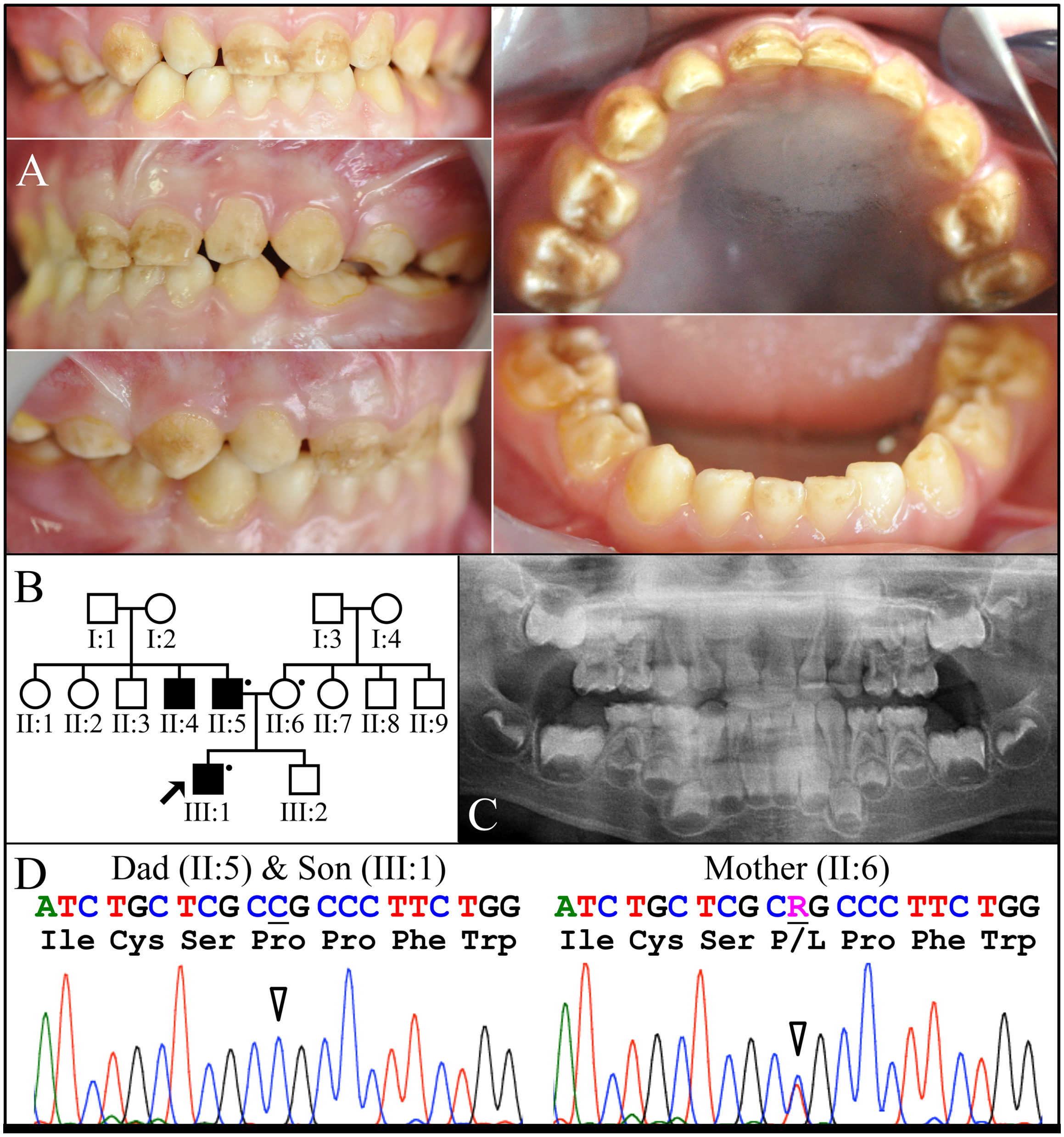

Figure S5 Family TKTO with AI associated with biallelic *GPR68* defects. *A:* Oral photographs of primary dentition in the male proband at age 5. His teeth exhibited a yellowish-brown discoloration with localized surface roughnesses and attrition of the enamel layer on working surfaces. The primary teeth were not widely separated (as often is the case with enamel hypoplasia) and the sizes of the dental crowns appeared to be within normal limits. *B:* Three generation pedigree. A dot marks each person who donated samples for DNA sequencing. *C:* Panoramic radiograph of the proband at age 5. The enamel does not contrast well with dentin in most places. Where the enamel does contrast, it appears to be thin. *D: GPR68* DNA sequence chromatograms from the proband (left) and mother (right). The father and the proband both were affected with AI and were homozygous for the *GPR68* sequence variation c.221T>C; p.(Leu74Pro) relative to the NCBI reverence sequence NM_001177676.1. The unaffected mother was heterozygous for this mutation.

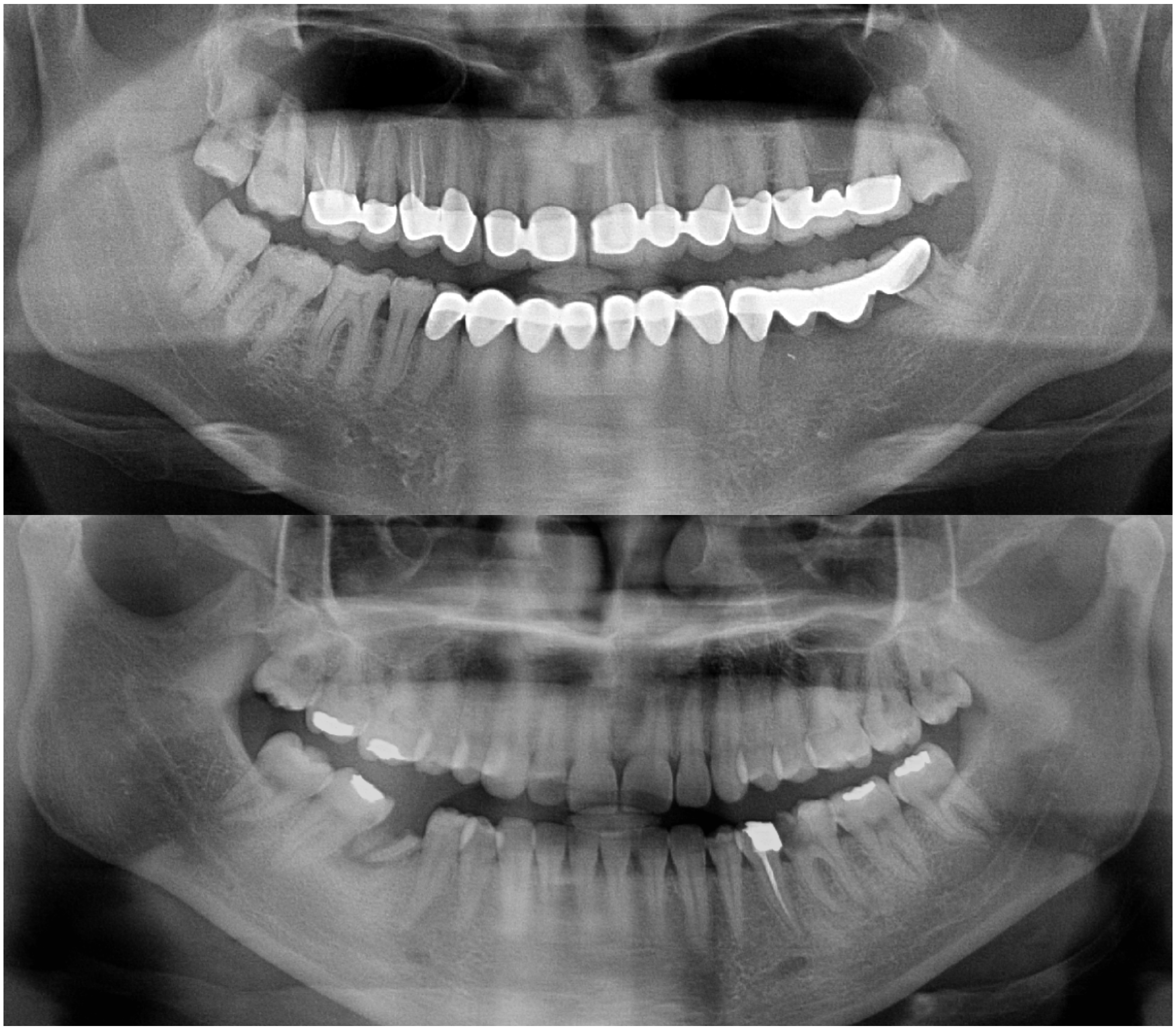

Figure S6. Radiographs from the parents of the proband in family TKTO. *A:* Panoramic radiograph of the affected father (II:5) of the proband (III:1) at age 29. The remaining dental enamel is thin. *B:* Panoramic radiograph of the unaffected mother (II:6) of the proband (III:1) at age 26. Her enamel is normal in thickness and contrast.

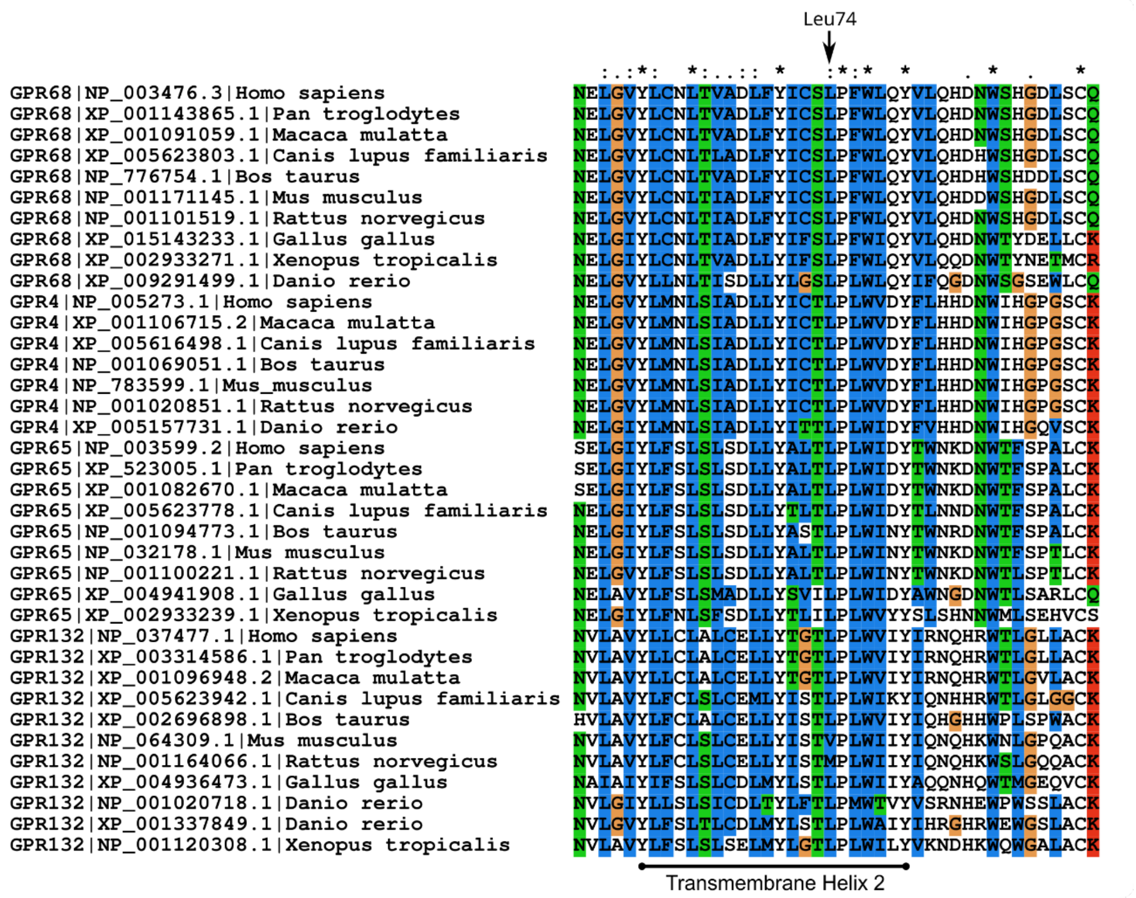

Figure S7. Conservation of GPR68 Leu74. A multiple sequence alignment of GPR68 orthologs and other proton-sensing G-protein coupled receptors was created using CLUSTALX. The position of GPR68 Leu74 is marked by an arrow above the alignment and the residues comprising the second transmembrane helix are marked by a bar underneath the alignment. Leu74 is completely conserved in the GPR68 orthologs identified and conserved in all proton-sensing G-protein coupled receptors except for mouse and rat GPR132, which show a conservative substitution to valine or methionine respectively.

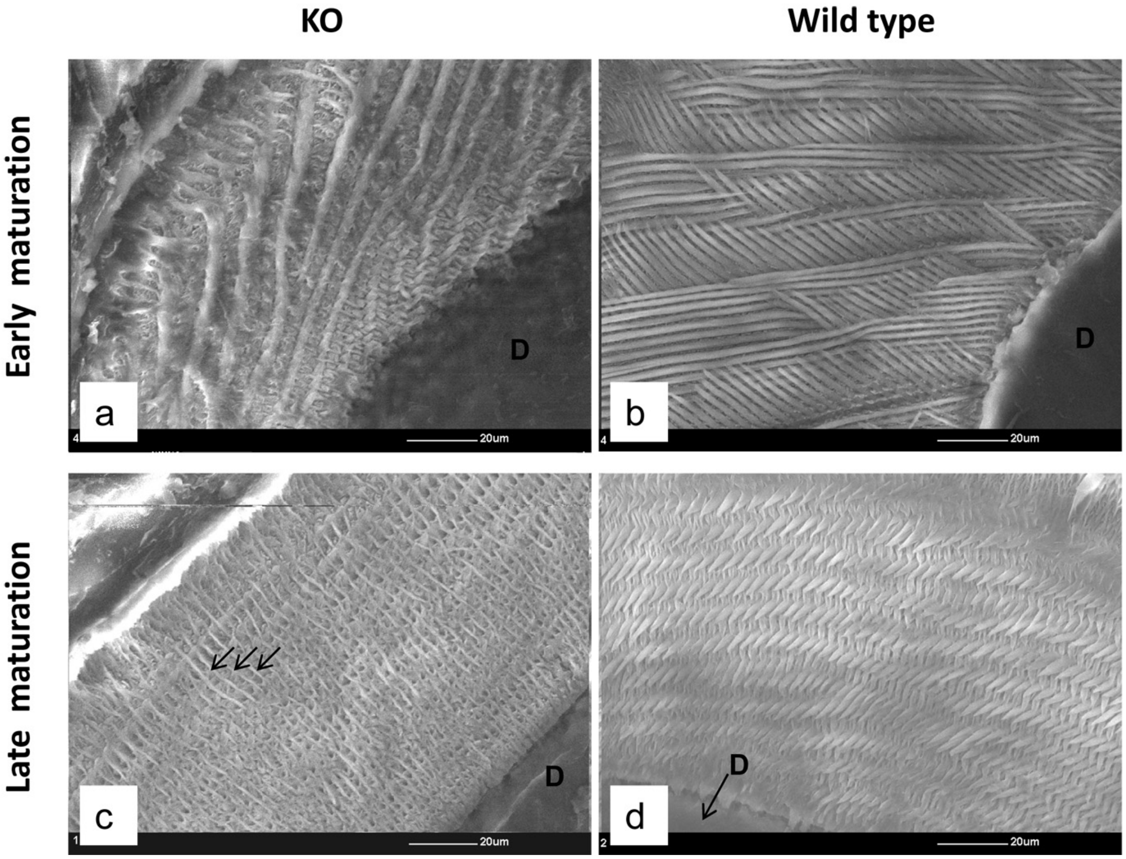

Figure S8. SEM appearance of *Gpr68* (*Ogr1*) KO and wild-type mouse incisor enamel. 'a' and 'b' are micrographs of KO and wild-type (WT) mouse incisor enamel respectively in the early stage of maturation (samples taken relative to landmarks within the mandible). SEM analysis indicated possible retardation in the formation of, and alteration in the structure of incisor enamel in knockout animals. In the WT, the characteristic decussating prism pattern can be seen whilst in the KO the appearance more resembles that usually seen in late secretion, indicating that the maturation process in the KO may be delayed. 'c' and 'd' are micrographs of KO and WT mouse incisor enamel in late maturation. By this stage, the KO seems to have 'rescued' the phenotype to a large degree, although subtle differences remain, with the interprismatic enamel (arrowed) appearing to be possibly more prominent in the KO compared to the WT. 'D' = dentine.

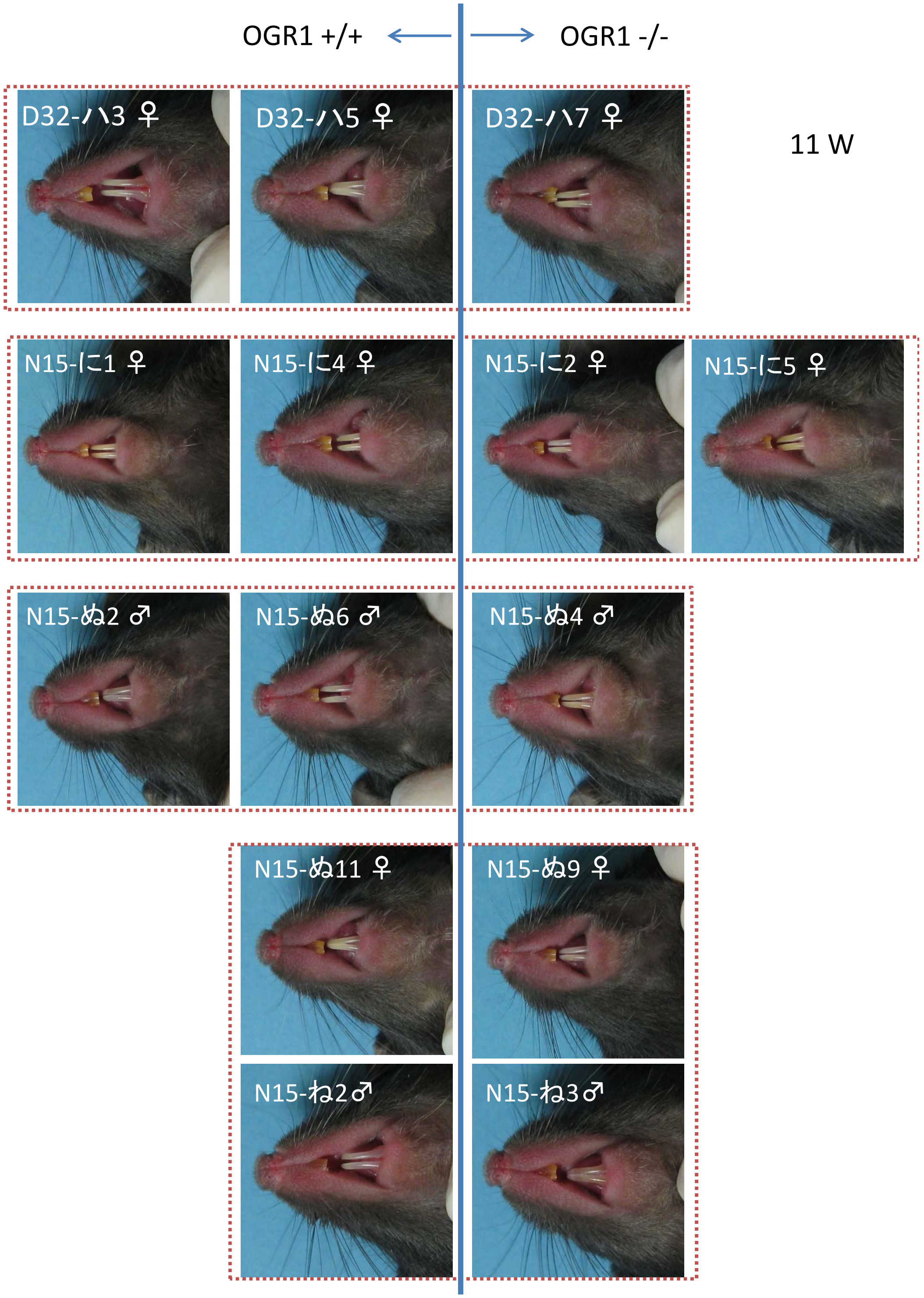
OGR1 +/+
OGR1 -/-
11 W
D32-ハ3 ♀
D32-ハ5 ♀
D32-ハ7 ♀
N15-に1 ♀
N15-に4 ♀
N15-に2 ♀
N15-に5 ♀
N15-ぬ2 ♂
N15-ぬ6 ♂
N15-ぬ4 ♂
N15-ぬ11 ♀
N15-ぬ9 ♀
N15-ね2♂
N15-ね3♂

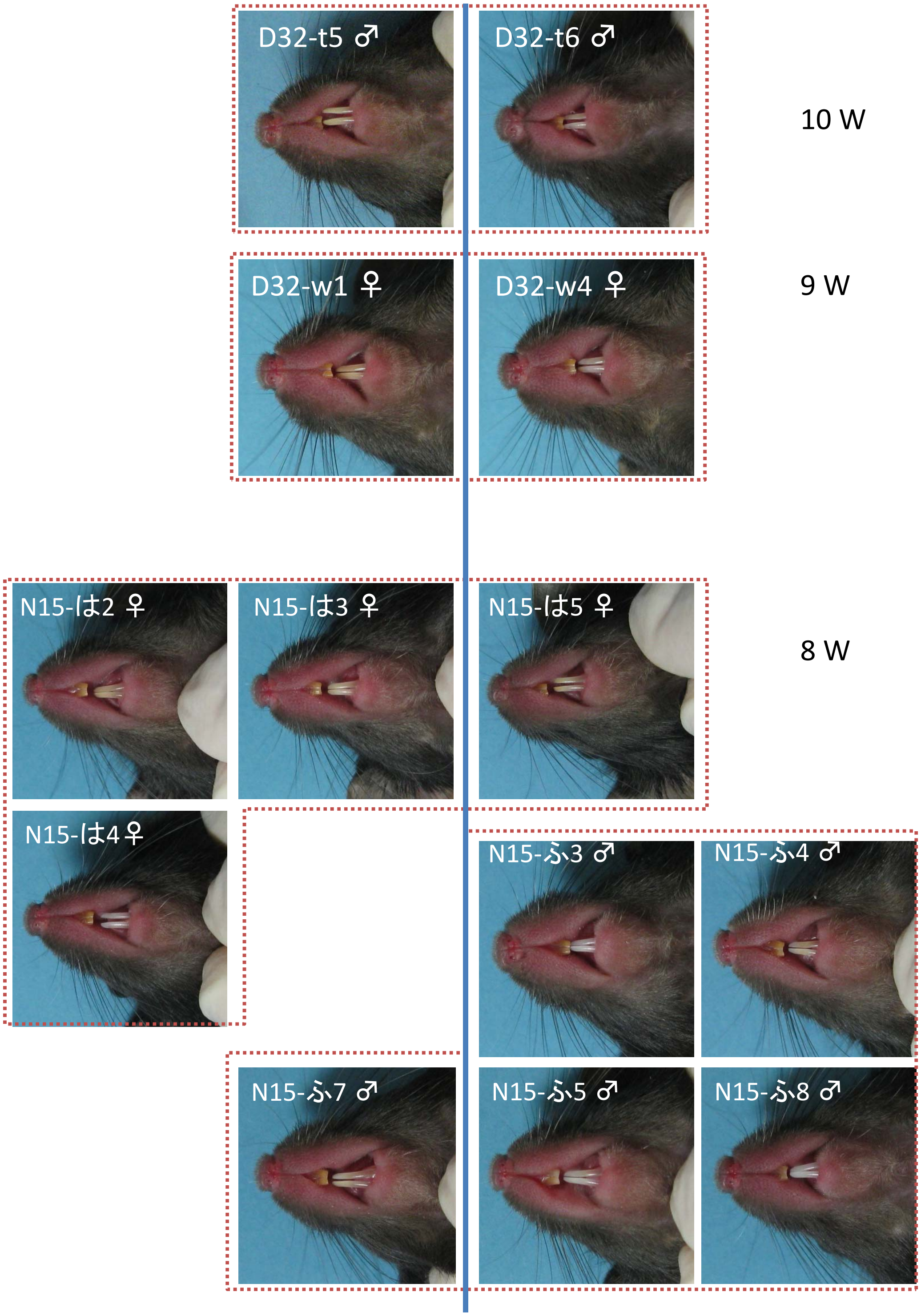

D32-t5 ♂
D32-t6 ♂
10 W
D32-w1 ♀
D32-w4 ♀
9 W
N15-は2 ♀
N15-は3 ♀
N15-は5 ♀
8 W
N15-は4♀
N15-ふ3 ♂
N15-ふ4 ♂
N15-ふ7 ♂
N15-ふ5 ♂
N15-ふ8 ♂

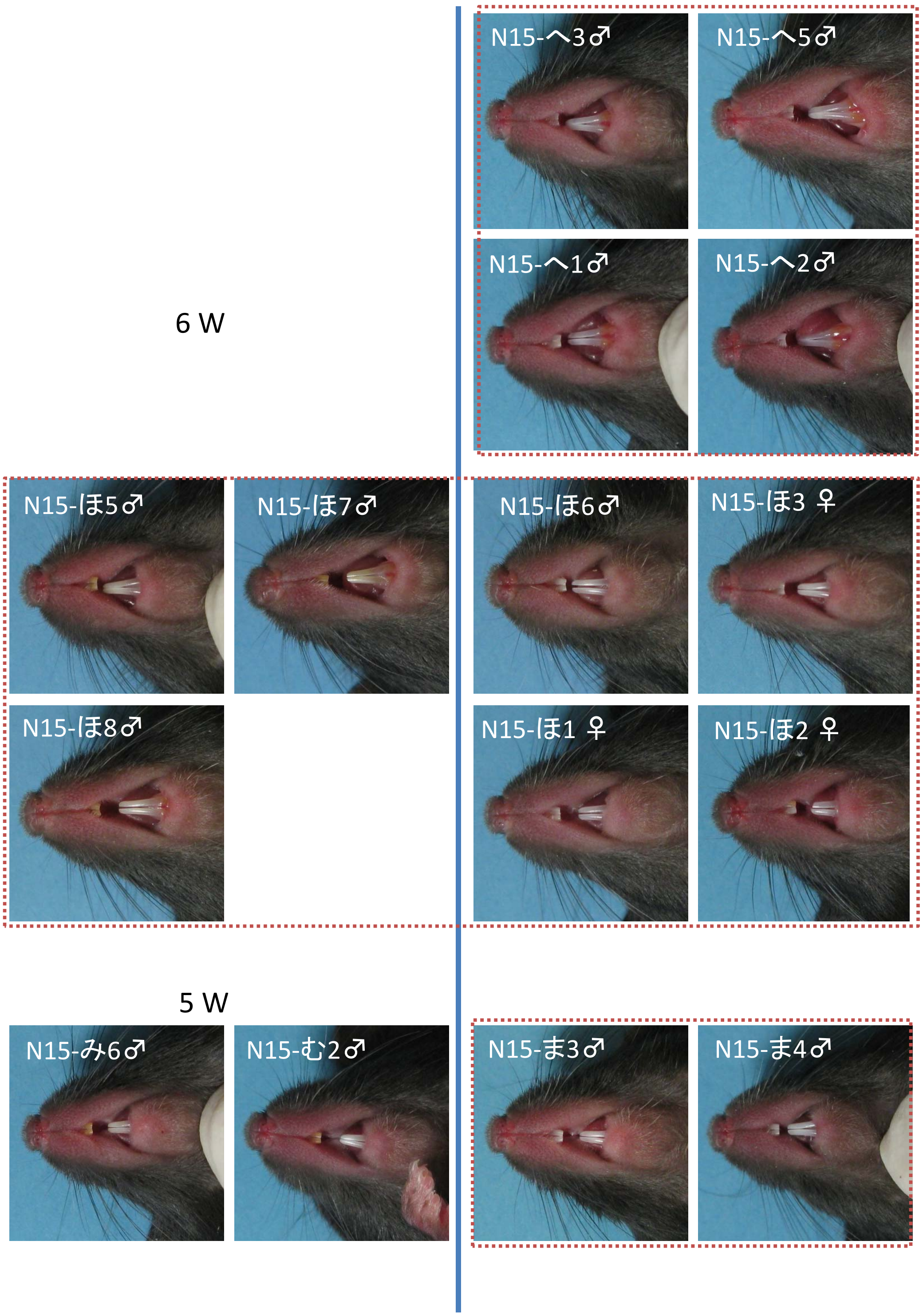
N15-へ3♂
N15-へ5♂
N15-へ1♂
N15-へ2♂
6 W
N15-ほ5♂
N15-ほ7♂
N15-ほ6♂
N15-ほ3 ♀
N15-ほ8♂
N15-ほ1 ♀
N15-ほ2 ♀
5 W
N15-み6♂
N15-む2♂
N15-ま3♂
N15-ま4♂

Figure S9. Appearances of incisor teeth of *Gpr68* (*Ogr1*) KO and wild-type mice at different ages between 5 and 11 weeks old. *Gpr68* (*Ogr1*) wild-type (WT) and KO mice can be found on the left and right hand side of the central solid blue line respectively. Litter mate mice are encircled by red dashed lines. When comparing the color of the maxillary (upper) incisors of WT and KO mice at 5 to 6 weeks, the incisors of KO mice remain white, where as those of WT are already yellow. By 8 weeks, the maxillary incisor teeth of the KO mice have the yellow coloring and are indistinguishable from WT mice.

Table S1. SNP genotypes for AI-5 individuals for the chromosome 14 locus. The disease region is highlighted in blue.

Table S2. Oligonucleotide primer pairs used to amplify and sequence the *GPR68*.

Amplicon	Forward	Reverse
Exon 2 part 1	CCTTTCCTGCCTCTGACTTTC	AGCACGTACTGCAGCCAGA
Exon 2 part 2	CTGCCTGTCCCTCTACTTCG	CAGATGGGGAAGAGGAAGC
Exon 2 part 3	GAGGTCATCGAGGACGAGAA	CTGGAGCAGGTGAGGAAGG
Exon 2 part 4	CCTGCTCACCAGCTTCAACT	CGGAGTTACAGGGGCTTCC
Full coding region	ACAAGGGCTTGCCATGTTAC	GCAGAGGACACGGCTTATTC
Confirmation of variant identified in family TKTO	CTGCCTGTCCCTCTACTTCG	CAGATGGGGAAGAGGAAGC

Genomic variant (GRCh37)	dbSNP	Predicted amino acid change	Gene	CADD v1.3[1]	SIFT[2]	Polyphen[3]	RefSeq transcript
14:89089048C>T	rs3681122776	p.G1638E	*EML5*	34	Deleterious (0)	Possibly damaging (0.721)	NM_183387
14:91700727delTT	N/A	p.K223Gfs*113	*GPR68*	33	N/A	N/A	NM_003485
7:141756708C>G	rs200326465	p.P1220R	*MGAM*	26.0	Deleterious (0)	Probably damaging (0.99)	NM_004668
6:144081721A>G	rs368546742	p.K202R	*PHACTR2*	24.2	Deleterious (0.01)	Probably damaging (0.986)	NM_014721
20:54823983C>A	N/A	p.N28K	*MC3R*	23.7	Tolerated (0.06)	Possibly damaging (0.643)	NM_019888
10:49459656G>C	N/A	p.S35C	*FRMPD2*	23.6	Tolerated (0.23)	Possibly damaging (0.907)	NM_001018071
6:32489744delG	N/A	p.A103*	*HLA-DRB5*	17.49	N/A	N/A	NM_002125

Table S3. Details of the 7 genomic variants identified in family AI-178 by exome sequencing after filtering. Exome sequencing was performed as described previously[4]. Homozygous variants present in IV:3 were selected and were filtered by removing those with a MAF of 1% or more in dbSNP142, EVS or ExAC[5]. The list is restricted to variants scoring 15 or more when scored with CADD v1.3[1].

Genomic variant (GRCh37)	dbSNP	Predicted amino acid change	Gene	CADD v1.1[1]	SIFT[2]	Polyphen[3]	RefSeq transcript
14:91701174A>G	N/A	p.L74P	*GPR68*	34	Damaging (0.001)	Possibly damaging (1)	NM_003485

Table S4. Detail of the homozygous variant identified in Family TKTO (II:5 and III:1). Homozygous variants were filtered by removing those with a MAF of 1% or more in dbSNP142, EVS or ExAC[5]. The list is restricted to variants scoring 15 or more when scored with CADD v1.1[1].

Web Resources

dbSNP, http://www.ncbi.nlm.nih.gov/projects/SNP/
Exome Variant Server, http://evs.gs.washington.edu/EVS/
ExAC, http://exac.broadinstitute.org
Combined Annotation Dependent Depletion,
http://cadd.gs.washington.edu/info
SIFT, http://sift.jcvi.org/
PolyPhen2 http://genetics.bwh.harvard.edu/pph2/

References

1. Kircher, M., Witten, D.M., Jain, P., O'Roak, B.J., Cooper, G.M., and Shendure, J. (2014). A general framework for estimating the relative pathogenicity of human genetic variants. Nat. Genet. *46*, 310–315.

2. Ng, P.C., and Henikoff, S. (2003). SIFT: Predicting amino acid changes that affect protein function. Nucleic Acids Res. *31*, 3812–3814.

3. Adzhubei, I.A., Schmidt, S., Peshkin, L., Ramensky, V.E., Gerasimova, A., Bork, P., Kondrashov, A.S., and Sunyaev, S.R. (2010). A method and server for predicting damaging missense mutations. Nat. Methods *7*, 248–249.

4. Logan, C. V, Cossins, J., Rodriguez Cruz, P.M., Parry, D.A., Maxwell, S., Martinez-Martinez, P., Riepsaame, J., Abdelhamed, Z.A., Lake, A.V.R., Moran, M., et al. (2015). Congenital Myasthenic Syndrome Type 19 Is Caused by Mutations in COL13A1, Encoding the Atypical Non-fibrillar Collagen Type XIII alpha1 Chain. Am. J. Hum. Genet. *97*, 878–885.

5. Consortium, E.A., Lek, M., Karczewski, K., Minikel, E., Samocha, K., Banks, E., Fennell, T., O'Donnell-Luria, A., Ware, J., Hill, A., et al. (2015). Analysis of protein-coding genetic variation in 60,706 humans (Cold Spring Harbor Labs Journals).
